# Association Between Kounis Syndrome and Allergy-Associated Coronary Events: A Disproportionality Analysis Using the Japanese Adverse Drug Event Report Database

**DOI:** 10.7759/cureus.90613

**Published:** 2025-08-20

**Authors:** Ichiro Nakakura, Yutaro Mukai, Atsuki Hosoda, Naohiro Ohara, Kaori Yamanishi, Takaya Uno, Satoshi Yokoyama, Kouichi Hosomi, Yoshiko Une

**Affiliations:** 1 Department of Pharmacy, National Cerebral and Cardiovascular Center, Suita, JPN; 2 Division of Drug Informatics, School of Pharmacy, Kindai University, Higashi-Osaka, JPN

**Keywords:** adverse event, allergic reaction, coronary artery disease, kounis syndrome, pharmacovigilance database

## Abstract

Background

Kounis syndrome (KS) is an allergy-induced acute coronary syndrome (ACS); however, the lack of established diagnostic criteria raises concerns regarding underdiagnosis. Misclassification of allergy-associated coronary events as non-KS conditions may hinder appropriate treatment and risk stratification. This study aimed to elucidate the clinical differences between KS and allergy-associated coronary events not diagnosed as KS (non-KS) to improve diagnostic recognition and clinical awareness.

Methods

Using the Japanese Adverse Drug Event Report database (2012Q3-2024Q2), we compared patient backgrounds, suspected drugs, and clinical features between the KS (n = 130) and non-KS (n = 115) groups. Multivariate analysis identified predictors of non-KS events.

Results

The KS group was associated with anesthetics and contrast media (e.g., iodinated agents such as iopamidol and iohexol), whereas the non-KS group was associated with heparin and the coronavirus disease 2019 vaccine. The non-KS group showed higher mortality and prevalence of specific underlying diseases than the KS group. Multivariate analysis identified heparin-induced thrombocytopenia (odds ratio (OR), 198.38; 95% confidence interval (CI), 39.45-3621.42), antineoplastic drug use (OR, 20.30; 95% CI, 6.68-76.87), fatal outcomes (OR, 11.98; 95% CI, 4.63-34.28), shock (OR, 10.02; 95% CI, 2.13-73.12), and ACS (OR, 5.66; 95% CI, 1.59-21.53) as independent predictors of non-KS events (all P < 0.05; area under the curve, 0.891).

Conclusions

Patients without KS often have severe underlying conditions and worse outcomes, suggesting a potential oversight of KS in clinical practice. These findings underscore the importance of improving the diagnostic clarity for KS and may contribute to future efforts to refine diagnostic criteria and pharmacovigilance practices.

## Introduction

Kounis syndrome (KS) is an acute coronary syndrome (ACS) triggered by allergic reactions, mediated by inflammatory mediators released from mast cells such as histamine, platelet-activating factor, arachidonic acid products, neutral proteases, and a variety of cytokines and chemokines [1, 2]. The clinical presentation of KS ranges in severity from unstable angina to life-threatening acute myocardial infarction and is characterized by coronary artery spasm or plaque erosion/rupture [1, 2]. KS can be induced by diverse factors, including drugs, environmental agents, foods, and coronary stents [1, 2].

In Japan, KS gained attention following the Ministry of Health, Labour and Welfare’s 2021 update to the precautions for sulbactam/cefoperazone, noting “acute coronary syndrome accompanying allergic reaction (KS),” and a special feature in the Pharmaceuticals and Medical Devices Agency (PMDA) Safety Information No. 387 [3].

The absence of standardized diagnostic criteria for KS has been identified as a major diagnostic challenge in previous reports [1, 2], contributing to underdiagnosis and frequent misclassification as anaphylaxis or unexplained ACS. Nevertheless, previous pharmacovigilance studies on KS have reported suspected drugs and safety signal strengths [4-6]. However, comprehensive analyses of potentially undiagnosed KS cases presenting with allergy-associated coronary events are limited. 

This study aimed to systematically investigate diagnosed and undiagnosed KS cases using the Japanese Adverse Drug Event Report (JADER) database to elucidate their clinical differences and highlight diagnostic challenges. 

## Materials and methods

Data source

This study used the JADER data provided by the PMDA, covering the period from Q3 2012 to Q2 2024. The JADER comprises four tables: patient demographics (demo), drug information (drug), adverse events (reac), and underlying diseases (hist). These tables were linked via identification numbers using Microsoft Power BI Desktop (Microsoft Corporation, Redmond, USA) to form a relational database. Owing to the nature of the JADER data, the complete removal of duplicate cases is challenging. Following a previous study [5] that prioritized data consistency and avoided analytical bias in large-scale pharmacovigilance databases by not removing duplicates, we retained duplicates to ensure consistency.

This study adhered to the READUS-PV (REporting of A Disproportionality analysis for drUg Safety signal detection using spontaneously reported adverse events in PharmacoVigilance) guidelines for conducting disproportionality analyses in pharmacovigilance [7], and followed the STROBE (Strengthening the Reporting of Observational Studies in Epidemiology) statement for reporting observational studies [8].

Study population and case selection

**Figure 1:**
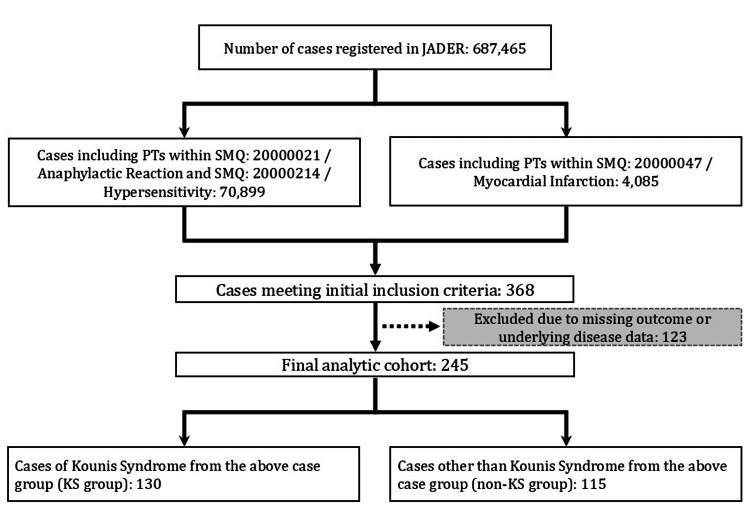
Flowchart of Case Selection Abbreviations: JADER, Japanese Adverse Drug Event Report Database; KS, Kounis syndrome; PT, Preferred Term; SMQ, standardized Medical Dictionary for Regulatory Activities queries Flowchart illustrating the selection process of Kounis syndrome and non-Kounis syndrome cases from the Japanese Adverse Drug Event Report database.

This study utilized data from the JADER database, covering reports from Q3 2012 to Q2 2024 (total: 687,465 cases). The overall case selection process is illustrated in Figure 1.

Adverse events were defined using Medical Dictionary for Regulatory Activities/Japanese version (MedDRA/J) version 27.1 [9], and patients were identified based on the following inclusion and exclusion criteria.

Inclusion Criteria

The inclusion criteria were: (a) Reported at least one Preferred Term (PT) under the Standardized MedDRA Query (SMQ) “Anaphylactic reaction” (SMQ code: 20000021) or “Hypersensitivity” (SMQ code: 20000214), and (b) Reported at least one PT under the SMQ “Myocardial infarction” (SMQ code: 20000047).

These SMQs were selected because they include the PT “Kounis Syndrome” (PT code: 10069167), enabling a comprehensive identification of Kounis syndrome cases and related concepts.

Exclusion Criteria

Cases with missing data on clinical outcomes or underlying diseases were excluded from the final analysis.

During the screening process, 70,899 cases met the inclusion criterion (a), and 4,085 cases met criterion (b). A total of 368 cases satisfied both criteria. Cases with missing data on clinical outcomes or underlying diseases were subsequently excluded (n=123).

The final analytical cohort consisted of 245 patients. This cohort was classified into two groups based on the presence or absence of the PT “Kounis Syndrome”: (1) KS group: Cases that included the PT “Kounis Syndrome” (n=130), and (2) Non-KS group: Cases that met the inclusion criteria but did not include the PT “Kounis Syndrome” (n=115) (Figure 1).

Data extraction and variable definitions

Data on suspected and concomitant drugs and patient characteristics, including sex, height, weight, age, outcomes, underlying diseases, and adverse events, were extracted. 

We first identified specific PTs reported in the KS group that fell under the allergic (SMQs “Anaphylactic reaction (20000021)” or “Hypersensitivity (20000214)”) and coronary (SMQ “Myocardial infarction (20000047)”) categories. These PTs were aggregated if they were reported alongside the PT-KS. We then assessed the presence of PTs in the non-KS group and aggregated the counts. 

Suspected and concomitant drugs were aggregated at the 5th and 2nd levels of the Anatomical Therapeutic Chemical (ATC) Classification System [10]. Drugs that were not classifiable at the ATC 5th level or lacked an ATC code were excluded. For drugs with multiple ATC 5th-level codes, aggregation was based on the name of the active ingredient. Drug use (suspected and concomitant) was aggregated at the ATC 2nd level. 

Age in JADER is recorded in 10-year categories. Given the increased risk of ACS in older adults, we categorized age into two groups: age <60 years vs. ≥60 years and <70 years vs. ≥70 years. 

Outcomes were classified into two categories based on the presence of death. Underlying diseases were assessed according to the definitions in Table 1 using MedDRA/J version 27.1 [9] (Table 1). Seven diseases were selected based on previous reports identifying them as risk or prognostic factors for KS or ACS [2, 11-13]. 

**Table 1:** Definitions of Underlying Diseases Abbreviations: HLGT, High-Level Group Term; HLT, High-Level Term; PT, Preferred Term; SOC, System Organ Class. The definitions are based on Medical Dictionary for Regulatory Activities/Japanese version (MedDRA/J) version 27.1 [9].

Underlying Disease	Definitions
Hypertension	Cases with PTs: Hypertension (10020772) and/or Essential hypertension (10015488)
Malignancy	Cases with PTs included in the SOC: Neoplasms benign, malignant and unspecified (incl cysts and polyps) (10029104), and also included under the High Level Group Term (HLGT): Malignant neoplasms (10010331).
Cardiac Disorder	Cases with PTs included in the SOC: Cardiac disorders (10007541).
Diabetes	Cases with PTs included in the HLT: Diabetes mellitus (including subtypes) (10012602).
Renal Impairment	Cases with any PT included in the HLT: Renal failure and impairment (10038443).
Allergy	Cases with PTs included in the SOC: Immune system disorders (10021428), under the HLGT: Allergic conditions (10001708).
Lipid Metabolism Disorder	Cases with PTs included in the SOC: Metabolism and nutrition disorders (10027433), under the HLGT: Lipid metabolism disorders (10013317).

Safety signal analysis for suspected drugs 

For the suspected drugs in the KS and non-KS groups, we calculated the reporting odds ratio (ROR), 95% confidence interval (CI), and Fisher’s exact test P-values as safety signal indicators. A safety signal was considered present if the lower limit of the 95% CI exceeded 1. For suspected drugs, ROR analysis was based on total counts derived from data aggregated at the ATC 5th level (ATC classification). ROR was calculated using a 2 × 2 contingency table using the formula: 

$ROR = \dfrac{(a/b)}{(c/d)} = \dfrac{(ad)}{(bc)}$　(Equation 1), 

Where $a$ = number of target adverse event reports for the target drug; $b$ = number of other adverse event reports for the target drug; $c$ = number of target adverse event reports for other drugs; and $d$ = number of other adverse event reports for other drugs.

To address zero cells, the Haldane-Anscombe correction was applied by adding 0.5 to each cell. 

For drugs with detected safety signals, we reviewed their Japanese electronic package inserts for references to “acute coronary syndrome accompanying allergic reaction” using the PMDA’s Medical Product Information Search [14]. 

Statistical analyses 

Univariate and multivariate logistic regression analyses were performed to compare the patient backgrounds, underlying diseases, and drug use between the KS and non-KS groups. These analyses also aimed to identify the independent predictors of non-KS classification. 

Univariate associations between each variable and group classification (KS vs. non-KS) were evaluated in the final cohort of cases eligible for multivariate analysis (i.e., those with no missing data for any candidate variable). For categorical variables, these associations were assessed using the chi-squared or Fisher’s exact test (for expected values <5). Variables with a P-value <0.05 in the univariate analysis were selected as candidates for the multivariate model. 

The selected candidate variables were then entered into a stepwise logistic regression model, with the non-KS group as the dependent variable. Multicollinearity was assessed using the variance inflation factor (VIF), and variables with a VIF of ≥5 were excluded. Odds ratios (ORs), 95% CIs, and area under the receiver operating characteristic curve (AUC) were calculated from the final model. Statistical significance was set at P < 0.05. 

For drug safety signal detection, the ROR, 95% CI, and Fisher’s exact test P-values were calculated. All ROR analyses were based on the aggregated data at the ATC 5th level. 

All statistical analyses were performed using R version 4.3.2 (R Foundation for Statistical Computing, Vienna, Austria) and JMP software version 9.0.2 (SAS Institute Inc., Cary, USA).

## Results

Suspected drugs 

A total of 58 suspected drugs were reported in the KS group, 33 of which had safety signals detected. The non-KS group reported 66 suspected drugs, with signals detected for 13 (Tables 2, 3). The only drugs to show safety signals in both groups were iopamidol and sugammadex sodium. In the KS group, safety signals were detected for the following drug categories: cardiovascular (e.g., epinephrine, nicorandil), antibacterials (e.g., cefazolin), anesthetics (e.g., propofol, lidocaine, remifentanil, sevoflurane), muscle relaxants (e.g., rocuronium), and analgesics (e.g., fentanyl, morphine). No safety signals were observed for any of these drugs in the non-KS group. By contrast, the non-KS group exhibited safety signals for heparin, the coronavirus disease 2019 (COVID-19) vaccines, and ipilimumab. There were no such signals in the KS group.

**Table 2:** Suspected Drugs With Safety Signals Detected in Kounis Syndrome Cases Abbreviations: ATC, Anatomical Therapeutic Chemical; ACS, acute coronary syndrome; CI, confidence interval; ePI, electronic package inserts; ROR, reporting odds ratio. Drug classification and codes are based on the Anatomical Therapeutic Chemical Classification System (2025) [10]. Drug names were aggregated at the ATC 5th level. ^†^P values were calculated using Fisher’s exact test. ^*^Iopamidol and sugammadex were also identified as suspected drugs with a detected safety signal in the non–Kounis syndrome group (see Table 3).

ATC code(s), Drug name	No. of cases	ROR	95% CI	P-value ^†^	ePI mention of "ACS with allergic reaction"
V08AB04 Iopamidol*	18	38.59	23.42–63.56	<0.001	+
V03AB35 Sugammadex*	15	69.31	40.36–119.04	<0.001	−
N01AX10 Propofol	12	13.15	7.26–23.83	<0.001	−
M03AC09 Rocuronium bromide	12	15.77	8.70–28.58	<0.001	−
N01AH01, N02AB03 Fentanyl	11	7.92	4.27–14.70	<0.001	−
J01DB04 Cefazolin	10	16.47	8.63–31.42	<0.001	+
V08AB02 Iohexol	7	26.59	12.39–57.04	<0.001	+
J01FA09 Clarithromycin	6	6.17	2.72–14.00	<0.001	−
A01AD01, B02BC09, C01CA24, R01AA14, R03AA01, S01EA01 Epinephrine	6	37.15	16.33–84.52	<0.001	−
N01AH06 Remifentanil	6	8.26	3.64–18.75	<0.001	−
C01BB01 Lidocaine	5	9.92	4.06–24.27	<0.001	−
C01DX16 Nicorandil	5	6.71	2.74–16.41	<0.001	−
C08CA15 Benidipine	4	6.93	2.56–18.77	0.003	−
J01DD17 Cefcapene	4	10.19	3.76–27.59	<0.001	−
V08AB10 Iomeprol	4	19.42	7.16–52.63	<0.001	+
J01DD62 Cefoperazone and beta-lactamase inhibitor (as Cefoperazone sodium and sulbactam sodium)	3	17.30	5.50–54.47	<0.001	+
J01DC09 Cefmetazole	2	7.86	1.94-31.79	0.028	−
N05CM18 Dexmedetomidine	2	5.81	1.44–23.48	0.048	−
V08AB05 Iopromide	2	37.80	9.31–153.52	0.002	−
N02AA01 Morphine	2	4.99	1.23–20.18	0.063	−
N02BA55 Salicylamide, combinations excluding psycholeptics	2	3579.85	593.17–21604.86	<0.001	−
N01AB08 Sevoflurane	2	5.34	1.32–21.58	0.056	−
J01DC04 Cefaclor	1	11.68	1.63–83.71	0.083	−
V08AA01 Diatrizoic acid	1	28.64	3.98–205.94	0.035	−
N05AD08 Droperidol	1	27.46	3.82–197.40	0.036	−
V08CA09 Gadobutrol	1	21.56	3.00–154.86	0.046	−
V08CA01 Gadopentetic acid	1	61.24	8.47–442.97	0.017	−
V03AB32 Glutathione	1	34.59	4.81–249.01	0.029	−
V04CX01 Indocyanine green	1	79.52	10.96–577.13	0.013	−
N01BB10 Levobupivacaine	1	13.62	1.90–97.67	0.071	−
N01BB03 Mepivacaine	1	16.19	2.26–116.14	0.061	−
V08DA06 Perflubutane, phospholipid microspheres	1	666.01	82.70–5363.42	0.002	−
J01CA12 Piperacillin	1	8.63	1.20–61.82	0.110	−

**Table 3:** Suspected Drugs With Safety Signals Detected in Non-Kounis Syndrome Cases Abbreviations: ATC, Anatomical Therapeutic Chemical; ACS, Acute Coronary Syndrome; CI: Confidence Interval; ePI: Electronic Package Inserts; ROR, Reporting Odds Ratio. Drug classification and codes are based on the Anatomical Therapeutic Chemical Classification System (2025) [10]. Drug names were aggregated at the ATC 5th level. ^†^P values were calculated using Fisher’s exact test. ^*^Iopamidol and sugammadex were also identified as suspected drugs with a detected safety signal in Kounis syndrome cases (see Table 2).

ATC code(s), Drug name	Number of cases	ROR	95% CI	P-value ^†^	ePI mention of "ACS with allergic reaction"
B01AB01 Heparin	41	56.15	38.31–82.30	<0.001	−
J07BN01 Covid-19, RNA-based vaccine	17	3.82	2.28–6.40	<0.001	−
L01FX04 Ipilimumab	6	2.65	1.17–6.03	0.031	−
V08AB04 Iopamidol*	5	10.86	4.43–26.64	<0.001	+
B02BX05 Eltrombopag	2	8.88	2.19–35.99	0.022	−
L04AX06 Pomalidomide	2	4.62	1.14–18.71	0.072	−
L01EX05 Regorafenib	2	4.94	1.22–20.00	0.064	−
V03AB35 Sugammadex*	2	9.31	2.30–37.73	0.021	−
L01ED03 Alectinib	1	9.59	1.34–68.79	0.100	−
L01EA04 Bosutinib	1	9.22	1.29–66.15	0.104	−
H05BX06 Evocalcet	1	10.37	1.45–74.37	0.093	−
S01JA01 Fluorescein	1	60.29	8.34–435.89	0.017	−
L03AB07 Interferon beta -1a	1	21.52	3.00–154.67	0.046	−

Patient characteristics, comorbidities, and outcomes

The final analytical cohort consisted of 245 patients, including 130 in the KS group and 115 in the non-KS group (Figure 1). Table 4 summarizes the available patient characteristics for both groups. Owing to missing data on sex, height, weight, and age, these variables were excluded from the group comparisons (Table 4). 

**Table 4:** Patient Characteristics Abbreviation: KS, Kounis syndrome Statistical comparisons between the two groups for patient characteristics were not conducted due to a substantial amount of missing data.

​ Characteristic	​ KS group (N=130)	​non-KS group (N=115)
Sex​	​	​
Male​	​95	75​
Female​	34​	40​
Unknown​	1	0​
Height​	​	​
130–139cm​	1	1
140–149cm​	1	5
150–159cm​	13	16
160–169cm​	18	20
170–179cm​	7	11
180–189cm​	1	0
Unknown​	89	62
Body weight​	​	​
30–39kg​	2	4
40–49kg​	3	11
50–59kg​	10	18
60–69kg​	14	16
70–79kg​	9	7
80–89kg​	2	1
90–99kg​	1	0
100–109kg​	1	1
Unknown​	88	57
Age​	​	​
10–19 years​	7	1
20–29 years​	2	2
30–39 years​	1	0
40–49 years​	2	9
50–59 years​	20	17
60–69 years​	33	34
70–79 years​	52	25
80–89 years​	12	19
≥90 years​	0	8
Unknown	1	0
≥60 years	97	86
≥70 years	64	52

Among the seven underlying diseases assessed, the non-KS group had a significantly higher prevalence of cardiac disorders (P = 0.013) and diabetes (P = 0.010) than the KS group (Table 5). In contrast, the KS group exhibited a significantly higher prevalence of allergic conditions (P = 0.004) than the non-KS group (Table 5). The mortality rate was also significantly higher in the non-KS group (P < 0.001) than in the KS group (Table 5). 

**Table 5:** Underlying Diseases and Fatal Outcomes Abbreviation: KS, Kounis syndrome ^†^P values were calculated using the chi-squared test with χ² statistic and degrees of freedom (df = 1) reported. P values <0.05 were considered statistically significant.

​Characteristic	​ KS group (N=130)	​non-KS group (N=115)	χ² (1)	P-value†
Underlying disease​	​	​		​
Hypertension​	31 (23.9%)​	38 (33.0%)​	2.55	0.110
Malignancy​	32 (24.6%)​	31 (27.0%)​	0.18	0.676​
Cardiac disorder​s	41 (31.5%)​	54 (47.0%)​	6.11	0.013
Diabetes​ mellitus	12 (9.2%)​	24 (20.9%)​	6.59	0.010
Renal failure and impairment	5 (3.9%)​	7 (6.1%)​	0.66	0.417
Allergy​ conditions	14 (10.8%)​	2 (1.7%)​	8.15	0.004
Lipid metabolism disorders	12 (9.2%)​	17 (14.8%)​	1.80	0.179
Cases including Death as an outcome​	7 (5.4%)​	37 (32.2%)​	29.73	<0.001

Drug use and adverse events 

Table 6 presents the categorization of drug use by the 2nd level of the ATC classification system. The KS group showed higher use of C01 (cardiac therapy), J01 (antibacterials for systemic use), N01 (anesthetics), N02 (analgesics), and V08 (contrast media) than the non-KS group. By contrast, the non-KS group exhibited greater use of B01 (antithrombotic agents) and L01 (antineoplastic agents). 

**Table 6:** Medication use by Pharmacological Class (ATC second level) Abbreviations: ATC, Anatomical Therapeutic Chemical; KS, Kounis syndrome; N/A, Not Applicable Data are presented as n (%). Medication use includes both suspected and concomitant drugs. Drug classification and codes are based on the Anatomical Therapeutic Chemical Classification System (2025) [10]. ^†^P values were calculated using the chi-squared test with χ² statistic and degrees of freedom (df = 1) reported, or Fisher's exact test where appropriate (no χ² statistic available). P values <0.05 were considered statistically significant.

ATC Code (ATC 2nd level name)	KS group (N=130)​	non-KS group (N=115)​	Test Statistic	P-value​†
B01 Antithrombotic agents​	19 (14.6%)	57 (49.6%)	χ² (1) = 34.84	<0.001​
C01 Cardiac therapy​	16 (12.3%)	3 (2.6%)	χ² (1) = 8.03	<0.001 ​
C07 Beta blocking agents​	6 (4.6%)	2 (1.7%)	Fisher's exact test, N/A	0.289
C09 Agents acting on the renin-angiotensin system​	1 (0.8%)	5 (4.4%)	Fisher's exact test, N/A	0.102​
J01 Antibacterials for systemic use​	34 (26.2%)	6 (5.2%)	χ² (1) = 19.6	<0.001​
L01 Antineoplastic agents​	4 (3.1%)	22 (19.1%)	χ² (1) = 16.60	<0.001​
L04 Immunosuppressants​	2 (1.5%)	0 (0%)	Fisher's exact test, N/A	0.500​
M01 Anti-inflammatory and antirheumatic products​	12 (9.2%)	4 (3.5%)	χ² (1) = 3.31	0.069​
N01 Anesthetics​	27 (20.8%)	2 (1.7%)	χ² (1) = 21.17	<0.001​
N02 Analgesics​	11 (8.5%)	3 (2.6%)	χ² (1) = 3.88	0.049​
V08 Contrast media​	34 (26.2%)	7 (6.1%)	χ² (1) = 17.62	<0.001​

Table 7 summarizes the frequency of major PTs associated with allergic reactions and coronary artery disease reported alongside PT-KS. As multiple PTs were reported per case, each PT was counted independently. In the KS group, 56 (43.1%) patients reported at least one of these PTs, compared with 45 (39.1%) patients in the non-KS group. On the other hand, the KS group reported a significantly higher rate of "Anaphylactic reaction (10002198)" (P = 0.017) compared to the non-KS group. The non-KS group had significantly higher reports of PT “Acute coronary syndrome (10051592)” (P = 0.009), PT “Heparin-induced thrombocytopenia (10062506)” (P < 0.001), PT “Rash (10037844)” (P = 0.005), and PT “Shock (10040560)” (P < 0.001) than the KS group. 

**Table 7:** Frequencies of selected Kounis syndrome-associated Preferred Terms in KS and non-KS groups Abbreviations: KS, Kounis syndrome; N/A; Not Applicable; PT, Preferred Term Counts represent the number of reported PTs; a single case may have multiple PTs reported.  Preferred Terms (PTs) are based on MedDRA/J version 27.1 [9]. ^†^P values were calculated using the chi-squared test with χ² statistic and degrees of freedom (df = 1) reported, or Fisher's exact test where appropriate (no χ² statistic available). P values <0.05 were considered statistically significant.

Preferred Term (PT)	KS group (N=130)​	non-KS group (N=115)​	Test Statistic	P-value^†^
PT: 10002388/Angina unstable	1	3	Fisher's exact test, N/A	0.344
PT: 10051592/Acute coronary syndrome	5	15	χ² (1) =6.88	0.009
PT: 10016029/Face oedema	1	2	Fisher's exact test, N/A	0.602
PT: 10058269/Troponin T increased	1	2	Fisher's exact test, N/A	0.602
PT: 10020751/Hypersensitivity	2	5	Fisher's exact test, N/A	0.188
PT: 10046735/Urticaria	2	0	Fisher's exact test, N/A	0.500
PT: 10013700/Drug hypersensitivity	1	1	Fisher's exact test, N/A	1.000
PT: 10037844/Rash	2	11	χ² (1) =7.83	0.005
PT: 10066973/Contrast media allergy	2	0	Fisher's exact test, N/A	0.500
PT: 10055048/Allergy to vaccine	1	0	Fisher's exact test, N/A	1.000
PT: 10062506/Heparin-induced thrombocytopenia	1	41	χ² (1) =52.30	<0.001
PT: 10040560/Shock	2	17	χ² (1) =14.95	<0.001
PT: 10002199/Anaphylactic shock	32	18	χ² (1) =3.02	0.082
PT: 10002198/Anaphylactic reaction	17	5	χ² (1) =5.70	0.017
PT: 10045240/Type I hypersensitivity	1	0	Fisher's exact test, N/A	1.000

Predictors of the non-Kounis syndrome group 

A univariate analysis identified 16 variables significantly associated with group classification (P < 0.05). These variables were subsequently selected as candidates for multivariate analysis (Tables 5, 6, 7). The final model was built using a stepwise multivariate logistic regression. No multicollinearity was detected, as all VIF values for the selected variables were low (range: 1.051-1.151). 

**Figure 2:**
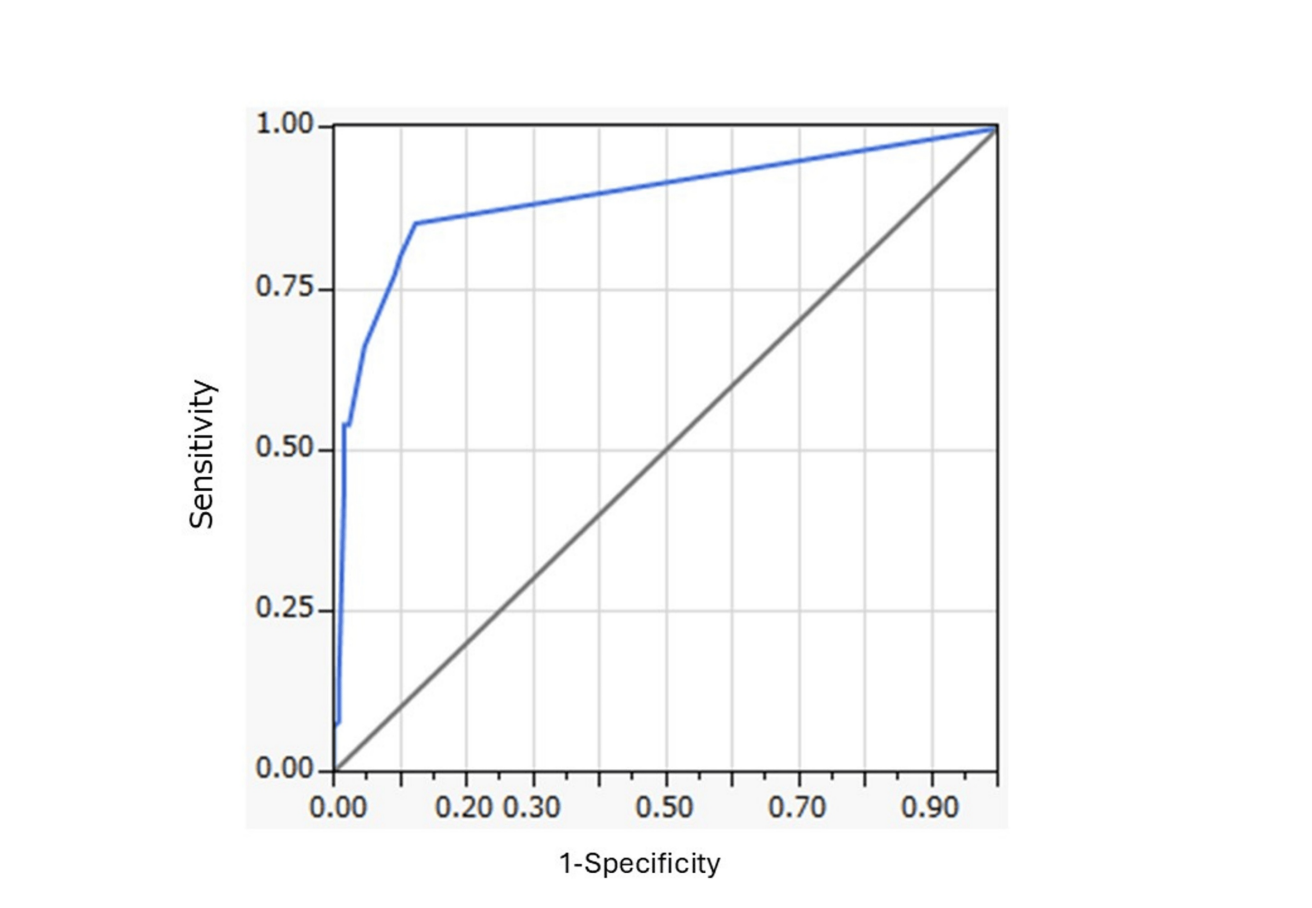
Receiver Operating Characteristic Curve of the Final Model Receiver Operating Characteristic (ROC) curve for the final multivariable logistic regression model predicting the non-KS (Kounis syndrome) group. The area under the ROC curve (AUC) was 0.891. The final model AUC used JMP software (version 9.0.2; SAS Institute Inc., Cary, NC, USA).

The final model identified the following five independent predictors of the non-KS group (Table 8): (1) PT “Heparin-induced thrombocytopenia”: OR 198.38 (95% CI, 39.45-3621.42; P < 0.001); (2) Use of L01 antineoplastic agents: OR 20.30 (95% CI, 6.68-76.87; P < 0.001); (3) Fatal outcome: OR 11.98 (95% CI, 4.63-34.28; P < 0.001); (4) PT “Shock”: OR 10.02 (95% CI, 2.13-73.12; P = 0.003); and (5) PT “Acute coronary syndrome”: OR 5.66 (95% CI, 1.59-21.53; P = 0.008). The model demonstrated strong predictive performance, with an AUC of 0.891 (Figure 2) and a pseudo R² of 0.454. Further model fit statistics are provided in Table 8. 

**Table 8:** Final Multivariable Logistic Regression Model for Predictors of Non-KS Group Abbreviations: AICc, Akaike Information Criterion corrected; AUC, Area under the receiver operating characteristic curve; BIC: Bayesian Information Criterion, CI: Confidence Interval, PT: Preferred Term, VIF: Variance Inflation Factor P values were calculated using the Wald test in a multivariable logistic regression analysis. P values <0.05 were considered statistically significant. Model fit statistics include Pseudo R², AICc, BIC, and AUC.

Variable	Odds Ratio (OR)	95% CI	Wald χ² (1)	P value	VIF
PT: Heparin-induced thrombocytopenia (10062506)	198.38	39.45–3621.42	25.77	<0.001	1.057
Use of L01 Antineoplastic agents​	20.30	6.68–76.87	24.34	<0.001	1.077
Fatal outcome	11.98	4.63–34.28	24.18	<0.001	1.113
PT: Shock (10040560)	10.02	2.13–73.12	7.13	0.003	1.151
PT: Acute coronary syndrome (10051592)	5.66	1.59–21.53	7.03	0.008	1.051
Model fit statistics					
Pseudo R²	0.454				
AICc	197.264				
BIC	217.919				
AUC	0.891				

## Discussion

This study compared the clinical characteristics of KS and allergy-associated coronary events that were not diagnosed as KS (non-KS) to highlight the diagnostic challenges and potential associations with KS. The key findings revealed significant differences in suspected drugs, patient backgrounds, underlying diseases, and clinical outcomes between the KS and non-KS groups. Notably, the non-KS group had a higher prevalence of specific underlying diseases and mortality than the KS group. This finding suggests that more severe conditions may be present in allergy-related coronary events that are not diagnosed as KS. 

In the KS group, drugs with prominent safety signals included anesthetics (e.g., propofol, rocuronium) and contrast media (e.g., iopamidol), consistent with known KS triggers [2, 4]. However, these drugs should be used with caution because of the risk of KS during perioperative or imaging procedures. Notably, iopamidol and sugammadex sodium showed safety signals in both groups. In Japan, iopamidol’s package insert includes a warning about “acute coronary syndrome with allergic reaction,” and it has been reported as a KS trigger [15, 16]. Sugammadex sodium is also known as a causative agent of anaphylactic shock and coronary artery spasm, and its association with Kounis syndrome has been reported [17]. These findings strongly suggest that iopamidol and sugammadex sodium are common risk factors for allergy-associated coronary events, including KS, regardless of whether KS was formally diagnosed. In contrast, the non-KS group showed safety signals for heparin, COVID-19 vaccines, and ipilimumab that were absent in the KS group.

Although heparin has been reported to trigger KS [2], it showed a safety signal only in the non-KS group. Multivariate analysis identified the Preferred Term heparin-induced thrombocytopenia (PT-HIT) as a strong predictor of non-KS (OR, 198.38), which is clinically notable. This suggests that HIT not only presents with ACS-like symptoms via thrombotic events but may also contribute to the pathophysiology of non-KS (allergy-associated ACS) through immunological mechanisms. Kounis et al. noted that HIT and KS shared a common pathophysiological mechanism of platelet activation despite having different triggers [18]. The non-KS group may include cases in which HIT and allergic reactions interact, resulting in overlapping clinical features and complex pathophysiology. Thus, in patients receiving heparin who present with allergic reactions and ACS, careful differential diagnosis that considers both HIT- and KS-like mechanisms is crucial. 

The detection of a safety signal for COVID-19 vaccines in the non-KS group highlights emerging concerns regarding vaccine-related adverse events. Although package inserts in Japan mention hypersensitivity and myocarditis/pericarditis, acute myocardial infarction or similar severe coronary events are not explicitly listed. However, various cardiovascular events have been reported following COVID-19 vaccination; in addition to the widely recognized myocarditis and pericarditis, few reports have documented acute myocardial infarction and similar severe coronary events, including KS [19, 20]. The safety signal for COVID-19 vaccines detected in our non-KS group suggests an association with vaccine-related cardiovascular adverse events.

Kounis et al. suggested that vaccine components, such as polyethylene glycol, might trigger immunoglobulin E-mediated anaphylaxis or complement activation, potentially causing KS [21]. Karimabad et al. discussed chemokines, such as the CXC motif chemokine ligand 10, which may be altered following vaccination and contribute to cardiovascular events [22]. However, a meta-analysis by Karimi et al. indicated that the benefits of vaccination outweighed the rare cardiovascular risks [23], suggesting that this signal should be interpreted as a rare event. Nonetheless, as highlighted by Nitz et al. [19], KS should be considered in the differential diagnosis of chest pain and allergic symptoms after vaccination. 

Multivariate analysis identified PT-ACS, PT-HIT, PT-Shock, fatal outcomes, and the use of L01 antineoplastic agents as independent predictors of non-KS. Although the association between PT-Shock and non-KS may appear counterintuitive, it likely reflects a limited awareness of KS and diagnostic uncertainty. In such cases, cardiovascular manifestations may overshadow allergic symptoms, resulting in the underdiagnosis or misclassification of KS. 

The identification of L01 antineoplastic agents' use as a predictor is noteworthy. Among antineoplastic agents with safety signals in the non-KS group, ipilimumab (an immune checkpoint inhibitor (ICI)) underscores the diagnostic challenge of differentiating ICI-related cardiovascular immune-related adverse events (irAEs) from drug-induced hypersensitivity reactions, such as KS, or recognizing their potential pathophysiological overlap. Antineoplastic agents cause hypersensitivity and cardiovascular toxicity [24]. Kounis et al. noted that chemotherapy and biologics could trigger KS via direct allergic reactions or cytokine release [25]. 

The non-KS group may include cases in which antineoplastic hypersensitivity or ICI-related irAEs (e.g., myocarditis, vasculitis) presented with ACS-like symptoms and allergic features but were not recognized as KS. Thus, the higher mortality in the non-KS group likely resulted from a combination of severe underlying diseases, more severe anaphylaxis, and the adverse prognostic impact of renin-angiotensin drugs, highlighting the need for increased clinical awareness to avoid missed KS diagnoses and ensure optimal interventions. 

Additionally, the non-KS group exhibited safety signals for protein kinase inhibitors (e.g., regorafenib, alectinib, bosutinib), which are associated with cardiovascular hypersensitivity reactions and other adverse events [26]. Their presence in this group may reflect either non-KS mechanisms of ACS misclassified as allergy-associated events or rare KS-like pathophysiology that went unrecognized. Although the limitations of self-reported databases preclude definitive pathophysiological conclusions, careful mechanistic evaluations are warranted when such agents induce cardiovascular events accompanied by allergic symptoms. 

Taken together, these findings underscore the importance of considering the diverse underlying mechanisms and performing careful differential diagnosis when allergic and ACS-like symptoms concurrently appear in patients receiving treatment with antineoplastic drugs, including ICI and some protein kinase inhibitors. 

The significantly higher mortality observed in the non-KS group may be attributable to a greater prevalence of cardiovascular risk factors, such as cardiac disorders and diabetes, which worsen ACS outcomes [11]. However, this alone does not fully explain the elevated mortality rate. The non-KS group had more cases of PT-Shock and cardiac disorders than the KS group. In drug-induced anaphylaxis, preexisting cardiovascular disease is a risk factor for severe or fatal outcomes [27]. Additionally, certain medications, such as beta-blockers, angiotensin-converting enzyme inhibitors/angiotensin II receptor blockers, and nonsteroidal anti-inflammatory drugs (NSAIDs), are associated with increased anaphylactic severity, treatment resistance, and mortality by impairing physiological compensation or reducing treatment efficacy (e.g., adrenaline) [28]. 

In this study, the use of beta-blockers (C07) did not significantly differ between the groups, and NSAID (M01) use was higher in the KS group than in the non-KS group; the use of renin-angiotensin system drugs (C09) was slightly more frequent in the non-KS group than in the KS group. These findings suggest that the elevated mortality rate in the non-KS group is unlikely to be attributable to the direct effects of beta-blockers or NSAIDs. Instead, the increased use of renin-angiotensin system drugs, which is consistent with the greater prevalence of cardiac disorders in this group, may reflect a more complex mechanism. In patients requiring these agents for underlying cardiovascular conditions, such medications may act as negative modifiers during severe anaphylaxis, impairing compensatory responses and worsening outcomes. 

Furthermore, in the non-KS group, the potential association between allergic reactions and cardiovascular events may have gone unrecognized, possibly resulting in suboptimal management of both anaphylaxis (e.g., delayed administration of adrenaline) and ACS (e.g., delayed catheterization). In KS-like scenarios involving severe anaphylaxis, clinical decision-making becomes more complex. 

Taken together, the higher mortality in the non-KS group likely reflects a multifactorial interplay of: (1) a higher burden of underlying disease, (2) more severe anaphylactic reactions, and (3) the potential of renin-angiotensin system drugs to exacerbate anaphylactic outcomes. These factors may contribute to treatment resistance and a poor prognosis. Missed KS diagnosis in high-risk patients can deprive them of optimal therapeutic interventions, underscoring the need for increased clinical awareness. 

Limitations

This study has some limitations. First, self-reported databases, such as JADER, are subject to reporting biases, including underreporting, selective reporting, and incomplete data. The exclusion of cases with missing information further limited the ability to draw causal inferences. Second, the lack of detailed clinical data (e.g., allergy testing, coronary angiography, treatment specifics) restricts the diagnostic accuracy and pathophysiological interpretation. Third, in the absence of standardized diagnostic criteria, the operational definitions used for the KS and non-KS groups may not fully reflect the underlying pathophysiology. Fourth, JADER does not provide information on the location of adverse events, which limits its ability to directly associate events with outcomes. 

Future directions

Future studies should focus on establishing internationally accepted diagnostic criteria for KS, developing diagnostic algorithms, and improving clinical education. Drugs identified with safety signals but lacking descriptions of “acute coronary syndrome accompanying allergic reaction” in their package inserts warrant further investigation and regulatory attention. Greater clinical awareness of allergy-associated coronary events that are not diagnosed as KS, as highlighted in this study, is essential for timely and appropriate management. Prospective, multicenter studies are required to clarify the true incidence, risk factors, pathophysiology, optimal treatment strategies, and long-term outcomes of KS. 

## Conclusions

Based on an analysis of the JADER database, this study identified notable clinical differences between the reported cases of the KS and non-KS groups involving allergy-associated coronary events. The non-KS group exhibited a higher burden of comorbidities and increased mortality than the KS group, raising the possibility that some KS cases may go unrecognized in clinical practice. These findings support the need for standardized diagnostic criteria for KS and greater clinical awareness to facilitate the timely recognition and appropriate management of allergy-associated cardiovascular events. 

## Appendices

Ethics approval and consent to participate 

This study used a publicly available database and did not involve identifiable personal data. Thus, per the National Cerebral and Cardiovascular Center, it is exempt from ethical review under the “Ethical Guidelines for Medical and Biological Research Involving Human Subjects.” Data usage complied with JADER’s terms of use. 
